# Laser Spectroscopy Measurements of Metastable Pionic Helium Atoms at Paul Scherrer Institute

**DOI:** 10.1007/s00601-021-01630-3

**Published:** 2021-07-29

**Authors:** M. Hori, H. Aghai-Khozani, A. Sótér, A. Dax, D. Barna

**Affiliations:** 1 Max-Planck-Institut für Quantenoptik, Hans-Kopfermann-Strasse 1, D-85748 Garching, Germany; 2 Present Address: McKinsey and Company, Munich, Germany; 3 Present Address: ETH Zürich, IPA, Zurich, Switzerland; 4 Present Address: Paul Scherrer Institut, CH-5232 Villigen, Switzerland; 5 CERN, CH-1211 Geneva, Switzerland; 6 Present Address: Institute for Particle and Nuclear Physics, Wigner Research Centre for Physics, Budapest, Hungary

## Abstract

We review recent experiments carried out by the PiHe collaboration of the Paul Scherrer Institute (PSI) that observed an infrared transition of three-body pionic helium atoms by laser spectroscopy. These measurements may lead to a precise determination of the charged pion mass, and complement experiments of antiprotonic helium atoms carried out at the new ELENA facility of CERN.

## Introduction

The PiHe collaboration recently carried out laser spectroscopy [1, 2] of metastable pionic helium ($\pi ^4\mathrm{He}^+\equiv {\pi ^-}+\mathrm{^4He}^{2+}+e^-$) using the 590 MeV ring cyclotron facility of PSI. This is a three-body atom [3–8] made of a $^4$He nucleus, an electron occupying the 1s ground state, and a negatively-charged pion in a state with principal and orbital angular momentum quantum numbers of $n\approx \ell +1\approx 17$. The Rydberg $\pi ^-$ orbitals have lifetimes of nanoseconds against the combined effects of $\pi ^-$ nuclear absorption, $\pi ^-\rightarrow \mu ^- +\overline{\nu }_{\mu }$ weak decay, and radiative and Auger decays. The orbitals have very small overlap with the nucleus so that strong interaction effects are negligibly small. The long lifetime allowed the first laser spectroscopy [2] of a mesonic atom to be carried out. Comparisons between the measured atomic frequencies and the results of three-body quantum electrodynamics (QED) calculations should in principle allow the $\pi ^-$ mass [9–11] to be precisely determined. Upper limits on laboratory constraints on the muon antineutrino mass [12], and possible exotic forces [13–16] involving $\pi ^-$ may also be set as in the case of metastable antiprotonic helium ($\overline{p}\mathrm{He}^+\equiv \overline{p}+\mathrm{He}^{2+}+e^-$) atoms [17–26]. Unlike normal atoms, $\pi ^4\mathrm{He}^+$ contains no hyperfine structure due to the spin-spin interaction between the spin-0 $\pi ^-$ and $^4$He nucleus. QED effects relevant to such boson-boson bound states can be studied with a high precision [27, 28].

Prior to this measurement, four experiments had observed that some $\pi ^-$ coming to rest in helium targets retain an anomalously long lifetime, thereby inferring the existence of $\pi \mathrm{^4He}^+$ [29–33]. Quantitative comparisons of the experimental data with theoretical calculations were difficult, however, as some sets of calculated decay rates of $\pi ^4\mathrm{He}^+$ states mutually differed by 1–2 orders of magnitude [1, 4, 5].

**Fig. 1:**
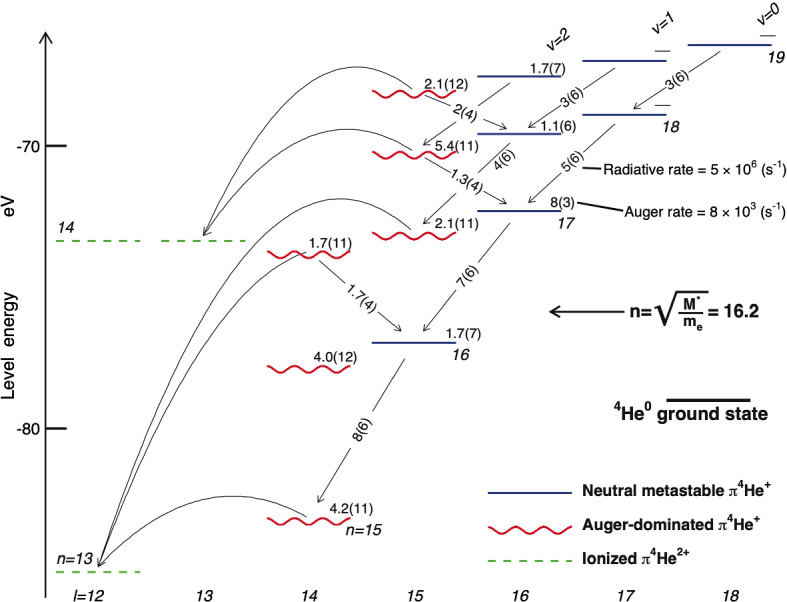
Energy level diagram of $\pi \mathrm{^4He}^+$ atoms. The theoretical energy of state $(n,\ell )$ is shown relative to the three-body-breakup threshold. The wavy lines indicate Auger-dominated states with picosecond-scale lifetimes, and the solid lines metastable levels with lifetimes of $>10$ ns. Auger decay rates of the states are indicated in s$^{-1}$. The dashed lines show the $\pi ^4\mathrm{He}^{2+}$ ionic states that are formed after Auger electron emission. The curved arrows indicate Auger transitions with minimum $|\Delta \ell _A|$. Radiative transitions $(n,\ell )\rightarrow (n-1,\ell -1)$ and $(n,\ell )\rightarrow (n-1,\ell +1)$ are shown by straight arrows, with the corresponding decay rates indicated in s$^{-1}$. From Ref. [1]

## Experimental Method

In the experiment, 800-ps long laser pulses of wavelength $\lambda \approx 1631$ nm excited a transition from a pionic state $(n,\ell )=(17,16)$ with a nanosecond-scale lifetime, to a resonance daughter state (17, 15) with a $\tau =5$ ps lifetime against Auger decay [1] (Fig. 1). The two-body $\pi \mathrm{^4He}^{2+}$ ion [34–37] that remained after Auger decay [38, 39] was destroyed in collisions with helium atoms. The laser resonance of $\pi \mathrm{^4He}^+$ was detected as a peak in the rate of neutrons, protons, and deuterons that emerged from the resulting $\pi ^-$ absorption. The signal was superimposed on a background of $\pi \mathrm{^4He}^+$ that decayed with a lifetime of $\tau \approx 7$ ns [1, 33].

For this experiment the $\pi$E5 beamline [40] of PSI produced a $\pi ^-$ beam of momentum $p=83$–87 MeV/c and intensity $N_{\pi }=(2-3)\times 10^7$ s$^{-1}$. A Wien filter and slit collimator removed most of the contaminant $e^-$ in the beam which had an intensity $>3\times 10^9$ s$^{-1}$. The purified $\pi ^-$ beam traversed a segmented plastic scintillator plate before entering the experimental helium target. The $\pi ^-$ arrived in bursts at intervals $\Delta t =19.75$ ns which corresponded to the $f_a=50.63$ MHz accelerating radiofrequency of the cyclotron. Each RF cycle contained on average $N_{\pi }/f_a\approx 0.4-0.6$ $\pi ^-$, which were distinguished from $\mu ^-$ and $e^-$ by the time-of-flight and energy loss in the scintillator plate.

**Fig. 2:**
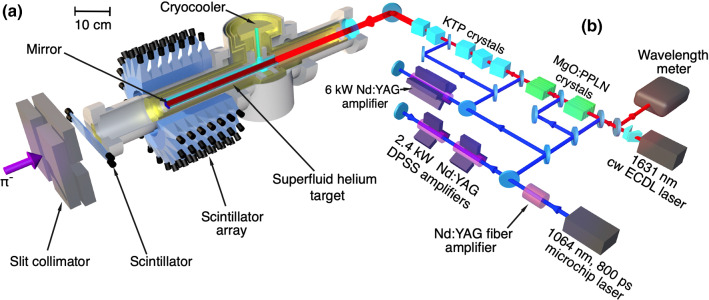
**a**: Layout of the experiment. The $\pi ^-$ beam traverses a segmented scintillation counter before coming to rest in the helium target, and the resulting metastable $\pi ^4\mathrm{He}^+$ atoms are irradiated with $\Delta t=800$ ps long laser pulses. The resulting neutrons, protons, and deuterons that emerge from the $\pi ^-$ absorption in the helium nuclei are detected by 140 plastic scintillation counters that surround the target. **b**: Schematic layout of the laser system. From Ref. [2]

The resonant laser pulses of energy $E=10$ mJ and repetition rate $f_r=80.1$ Hz were generated [2] by an injection-seeded, optical parameteric generator (OPG) and amplifier (OPA) laser system which were based on magnesium oxide doped periodically-polled lithium niobate (MgO:PPLN) and potassium titanyl phosphate (KTP) crystals (see Fig. 2 (b)). The linewidth of the narrowband component of the laser excluding the amplified spontaneous emission (ASE) was around $\approx 10$ GHz. A 3 GHz uncertainty in the optical frequency of the laser pulses was introduced by the OPG and OPA processes.

The laser beam of diameter $d=25$ mm entered the target chamber and irradiated $>60\%$ of the $\pi ^4\mathrm{He}^+$ atoms at a time $t=9$ ns after $\pi ^-$ arrival. We assumed that about 2.3$\%$ [33] of the $\pi ^-$ that stopped in the superfluid helium target (Fig. 2 (a)) formed the long-lived atoms. The estimated production rate $>3\times 10^5$ s$^{-1}$ of the atoms ensured that the probability of coincidence of a laser pulse irradiating an atom would be around $10^{-3}$.

The neutrons, protons, deuterons, and tritons that emerged from the $\pi ^-$ absorptions tended to follow anticollinear [1, 41, 42] trajectories and had kinetic energies of a few tens of MeV. The arrival times and energy depositions of these nuclear fragments were measured by an array of 140 plastic scintillation counters that covered a solid angle of $\approx 2\pi$ steradians around the target. The size $40\times 35\times 34$ mm$^3$ of the counters provided a $<10\%$ detection efficiency for signal $E\ge 25$ MeV neutrons [1]. We rejected most of the background $e^-$ that either arrived in the particle beam or were produced by $\mu ^-$ decays by removing events with small energy depositions. The waveforms [43–47] of the signals from the scintillation counters were recorded using some data acquisition electronics developed by us. A prototype was used earlier in an experiment to determine the limits on the annihilation cross sections $\sigma _A$ of antiprotons of kinetic energy $E\approx 125$ keV in thin target foils [46, 48, 49]. The results were compared with other measurements of $\sigma _A$ for antiprotons of energy $E=5.3$ MeV [47, 50–52].

## Experimental Results

**Fig. 3:**
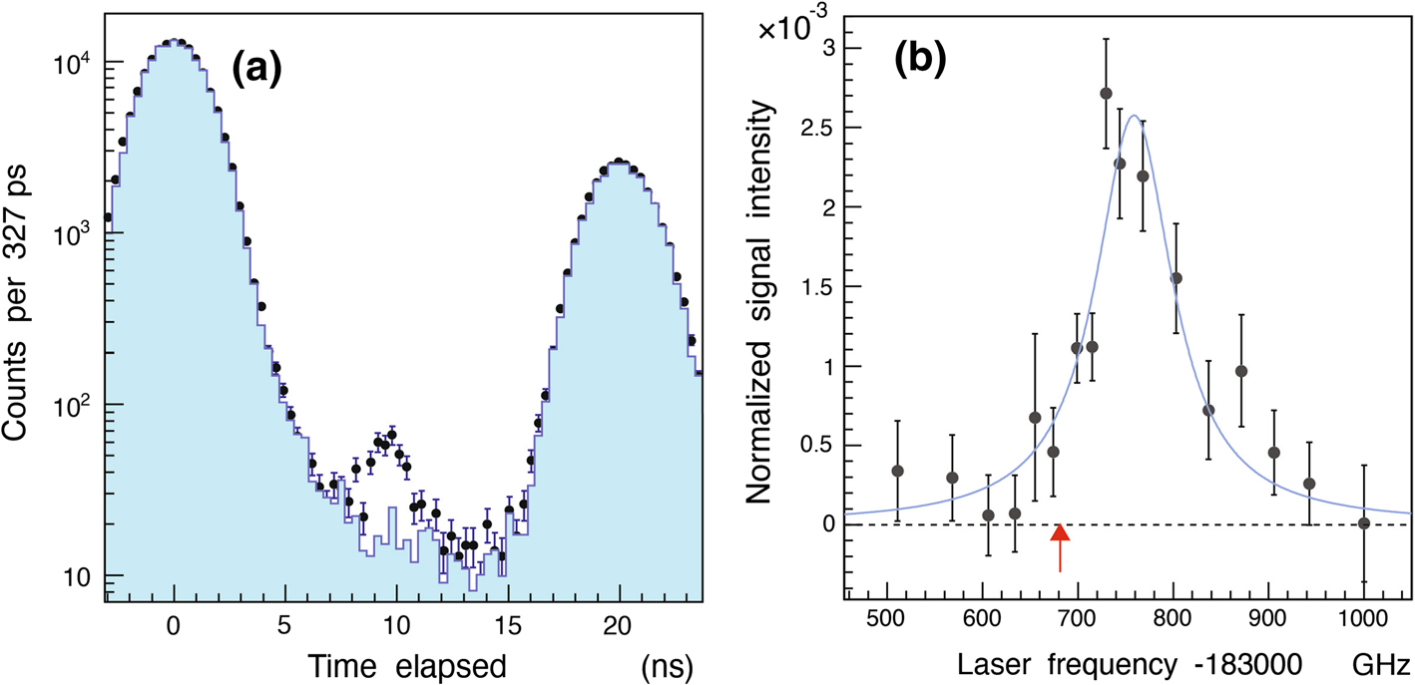
**a**: Time spectra of nuclear fragments measured with (indicated by filled circles with error bars) and without (blue filled histogram) laser irradiation at time $t=9$ ns. The peak in the former spectrum at $t=9$ ns corresponds to the resonance signal of $(17,16)\rightarrow (17,15)$. **b**: Profile of the resonance measured by scanning the laser frequency over a 500 GHz wide region and plotting the normalized counts under the peaks. From Ref. [2]

The blue time spectrum in Fig. 3 (a) shows the distribution of scintillator arrivals measured without laser irradiation. The peaks at $t=0$ and 19.75 ns correspond to consecutive $\pi ^-$ arrivals and contain the $>97\%$ majority of $\pi ^-$ that underwent immediate nuclear absorption. The remaining $(2.1\pm 0.7)\%$ produced a spectrum of the long-lived $\pi \mathrm{^4He}^+$ that decayed with a lifetime $\tau =(7\pm 2)$ ns in the intervals between the $\pi ^-$ arrivals. This spectrum roughly agreed with the expected signal according to a Monte Carlo simulation [1], and with the results of a previous experiment [33] carried out using a liquid helium target.

We searched for the transition $(n,l)=(16,15)\rightarrow (17,14)$ using a combined dye and Ti:Sapphire [53] laser to scan over a 200 GHz region around the theoretical transition frequency $\nu _\mathrm{th}=781052.6(2.0)$ GHz [1], but no significant signal was observed. Calculations show that the daughter state (17, 14) of this resonance couples to an electronically-excited $\pi ^4\mathrm{He}^+$ state which leads to large polarizabilities [6] that destabilize the state against atomic collisions. We also unsuccessfully searched for the $(16,15)\rightarrow (16,14)$ resonance which is expected to have a large width $\Gamma _A=640$ GHz. The reason for the non-observation is not understood, but atomic collisions may destroy the population in the resonance parent state (16, 15). Similar effects have been observed in several $\overline{p}\mathrm{He}^+$ states [54, 55]. An alternative explanation is that the state (16, 15) is not populated during the formation of the atom [24, 56–60]. Theoretical calculations of the formation process involve solving the dynamics of a four-body system and are complicated.

We then searched for the transition $(17,16)\rightarrow (17,15)$. The time spectrum shown using filled circles in Fig. 3 (b) represents data collected from $2.5\times 10^7$ $\pi ^-$ arrivals, with the OPG laser wavelength tuned to $\lambda \approx 1631.4$ nm. We consequently detected a peak at time $t=9$ ns which contained 300 events with a signal-to-noise ratio of 4 and a statistical significance of $>7$ standard deviations. The experimental detection rate of 3 h$^{-1}$ resonant $\pi ^4\mathrm{He}^+$ events is compatible with the implied production rate $>3\times 10^5$ s$^{-1}$ of the atoms, and with Monte Carlo simulations [1] that assume that most of the metastable pionic population occupies the parent state (17, 16). The signal decreased and disappeared as expected when the laser was detuned off the resonance frequency.

The resonance profile shown in Fig. 3(b) was obtained by scanning the laser frequency and plotting the number of arrival events under the peak induced by the laser. Each data point contains experimental data collected over a 20–30 h period. The vertical error bars indicate the statistical uncertainty arising from the finite numbers of the resonant $\pi \mathrm{^4He}^+$ events. The $\approx 100$ GHz width of the observed resonance is consistent with a convolution of the Auger width $\Gamma _A=33$ GHz of state (17, 15) [1] , collisional [7] and power broadening ($\approx 50$ GHz) effects, and the linewidth ($\approx 10$ GHz) of the narrowband component of the OPG laser pulses. Atomic collisions that shorten [6, 54] the lifetime of the daughter state (17, 15) may cause additional broadening of the resonance. The 3.0 GHz spacing [1] between the fine structure sublines that arise from the interaction between the electron spin and the orbital angular momentum of $\pi ^-$ is much smaller than the 33 GHz natural width of the resonance and so cannot be resolved. The best fit (blue curve) of two overlapping Lorentzian functions which take these hyperfine sublines into account had a reduced $\chi ^2$ value of 1.0, with a resonance centroid $\nu _\mathrm{exp}=183760(6)(6)$ GHz. The statistical uncertainty of 6 GHz here arises from the finite number of detected $\pi \mathrm{^4He}^+$, whereas the systematic uncertainty of 6 GHz contains contributions related to the selection of the fit function (5 GHz), the calibration of the laser frequency, and the uncertainty related to the OPG and OPA laser processes (3 GHz).

The experimental transition frequency is larger than the calculated frequency [1] $\nu _\mathrm{th}=(183681.8\pm 0.5)$ GHz. This $\Delta \nu =(78\pm 8)$ GHz deviation is believed to be due to atomic collisions in the experimental target that shift the resonance frequency [6, 7]. Collisional shifts of similar magnitude have previously been observed [54, 61] for some laser resonances of $\overline{p}\mathrm{He}^+$. The gradient of this shift in targets of temperature $T=4$ K was calculated as $d\nu /d\rho =(4.4-6.5)\times 10^{-21}$ GHz$\cdot$cm$^3$ using the impact approximation of the binary collision theory of spectral lineshapes [7]. At the superfluid target density $\rho =2.18\times 10^{22}$ cm$^{-3}$ used in these experiments, the predicted blueshift corresponds to a value between $\Delta \nu =96$ and 142 GHz, which roughly agrees with the experimental result.

In the future we will search for other laser transitions such as $(n,l)=(17,16)\rightarrow (16,15)$, which is predicted to be narrower by a factor of at least $10^{-3}$ compared to the transition $(17,16)\rightarrow (17,15)$ which was recently detected [1]. The experiments will be carried out using gas targets so that the collisional shifts will be smaller. The precision of the theoretical transition frequency $\nu _\mathrm{th}$ is now limited by the experimental uncertainty of the $\pi ^-$ mass. The precision of the calculations themselves [1], however, can be improved to better than $10^{-8}$ for some transitions as in the HD$^+$ [62–64] and $\overline{p}\mathrm{He}^+$ [17, 18] cases. These experiments will complement two-photon laser spectroscopy measurements on $\overline{p}\mathrm{He}^+$ [65] carried out at the new ELENA facility [66, 67] of CERN.
